# Deployment of attention to facial expressions varies as a function of emotional quality—but not in alexithymic individuals

**DOI:** 10.3389/fpsyt.2024.1338194

**Published:** 2024-03-06

**Authors:** Chiara Surber, Dennis Hoepfel, Vivien Günther, Anette Kersting, Michael Rufer, Thomas Suslow, Charlott Maria Bodenschatz

**Affiliations:** ^1^ Department of Psychosomatic Medicine and Psychotherapy, University of Leipzig Medical Center, Leipzig, Germany; ^2^ Department of Psychiatry, Psychotherapy and Psychosomatics, Psychiatric University Hospital Zurich (PUK), University of Zurich, Zurich, Switzerland; ^3^ Center for Psychiatry and Psychotherapy, Clinic Zugersee, Triaplus AG, Zug, Switzerland

**Keywords:** alexithymia, eye-tracking, facial emotions, lexical emotional priming, attentional preference, positive bias, gaze behavior

## Abstract

**Background:**

Alexithymia is a risk factor for emotional disorders and is characterized by differences in automatic and controlled emotion processing. The multi-stimulus free-viewing task has been used to detect increased negative and reduced positive attentional biases in depression and anxiety. In the present eye-tracking study, we examined whether lexical emotional priming directs attention toward emotion-congruent facial expressions and whether alexithymia is related to impairments in lexical priming and spontaneous attention deployment during multiple face perception.

**Materials and methods:**

A free-viewing task with happy, fearful, angry, and neutral faces shown simultaneously was administered to 32 alexithymic and 46 non-alexithymic individuals along with measures of negative affect and intelligence. Face presentation was preceded by masked emotion words. Indices of initial orienting and maintenance of attention were analyzed as a function of prime or target category and study group.

**Results:**

Time to first fixation was not affected by prime category or study group. Analysis of fixation duration yielded a three-way interaction. Alexithymic individuals exhibited no prime or target category effect, whereas non-alexithymic individuals showed a main effect of target condition, fixating happy faces longer than neutral and angry faces and fearful faces longer than angry faces.

**Discussion:**

Our results show evidence of attentional biases for positive and fearful social information in non-alexithymic individuals, but not in alexithymic individuals. The lack of spontaneous attentional preference for these social stimuli in alexithymia might contribute to a vulnerability for developing emotional disorders. Our data also suggest that briefly presented emotion words may not facilitate gaze orientation toward emotion-congruent stimuli.

## Introduction

The concept of alexithymia was introduced in the early 1970s (1) and refers to differences in the cognitive processing of emotion: difficulties in identifying and distinguishing feelings from bodily sensations, difficulties in describing feelings, and an externally oriented thinking style (2). The prevalence of alexithymia in the general population is estimated at approximately 10% (3–5), with a slightly higher frequency in men (4, 6, 7). Alexithymia is assumed to be a vulnerability factor for the development and maintenance of mental disorders such as depressive and anxiety disorders (3, 4, 8). Approximately half of the individuals with autism spectrum disorders experience co-occurring alexithymia (9). Emotion-processing difficulties in autism could be rooted to a large extent in alexithymia (10, 11). There is evidence that alexithymia is linked to increased negative affectivity (e.g., trait anxiety and depressive symptoms) (12, 13). It has been argued that people with alexithymia have less differentiated and accurate perceptions of their emotions upon which to base emotion regulation decisions (14). As a consequence of these differences, alexithymic individuals could be less effective at downregulating negative emotions. Moreover, experiences of negative emotions could be more frequent or prolonged by their use of less efficient emotion regulation strategies, i.e., more suppression and less reappraisal (15).

Alexithymia is characterized by multifaceted impairments in processing external emotional information (16). Individuals with alexithymia have problems in identifying other people’s emotional facial expressions at controlled (17) and automatic processing levels (18, 19). Alexithymia is also associated with a reduced capacity to recognize emotions from vocal expressions (20, 21) or lexical stimuli (22, 23). The emotion-processing impairments in alexithymia not only could consist of modality- or domain-specific (e.g., face-specific) differences in emotion recognition but also could extend to problems in the integration of emotional information across processing domains (24, 25). Decreased attention allocation to emotional stimuli during early processing could be one mechanism underlying the differences in emotion recognition in alexithymia (16).

The eye-tracking methodology has become an important tool in clinical studies to better understand attention allocation to emotional stimuli (26, 27). Eye tracking provides a rather direct measure of attention allocation, as the focus of attention and the direction of gaze appear tightly coupled (28). The multi-stimulus free-viewing task (based on pictorial, facial, or lexical stimuli) constitutes a frequently used eye-tracking paradigm that instructs participants to observe images freely with no constraints on attention (29). Free-viewing tasks provide naturalistic estimates of early and late processes of attention allocation, e.g., indices of initial orienting to or maintenance of attention on specific stimulus categories (30). In the last decades, research based on the free-viewing task has substantially contributed to detecting anxiety- and depression-related impairments in attention. Results from recent meta-analyses have indicated associations of anxiety with reflexive orienting toward and maintenance of attention on threat (30) and associations of depression with diminished sustained attention on positive stimuli and increased attention maintenance on dysphoric stimuli (31).

In multi-stimulus free-viewing tasks, individuals typically exhibit an attentional bias favoring positive stimuli (32–34), which is assumed to have a mood-protective function (35, 36). An attentional bias to positive information may protect against negative emotional responses to stressors and increase reward perception (37, 38) and can constitute an adaptive part of antecedent emotion regulation, which operates before a negative emotional experience is elicited *via* the production of positive emotional feelings (39). However, attentional biases to negative, fear-related stimuli have also been reported (40, 41). Such biases are also common and not necessarily maladaptive as they facilitate threat perception and immediate defensive responses (42). Free-viewing studies using face pairs provided evidence for attentional bias toward fearful faces (43, 44).

To our knowledge, there are three eye-tracking studies in which free-viewing tasks were administered to examine the effects of alexithymia on attention allocation to emotional information (45–47). In one investigation, a cued viewing task was administered (48). In all free-viewing studies, emotional images (scenarios) served as stimulus material. Overall, the findings of these investigations are rather inconsistent and suggest that specific facets of alexithymia might go along with the late avoidance of sad stimuli (47) but the early avoidance of and late sustained attention toward fear stimuli (46). None of these studies investigated the influence of high levels of alexithymia on attention to emotional information compared with non-alexithymia. Such studies would be important because some impairments in attention allocation could become apparent only in high alexithymia. Moreover, in the aforementioned studies, facial expressions were not administered as stimuli despite facial emotions being known to be prioritized during perception because of their adaptive and social significance (49–51), and alexithymia was found to be linked to differences in facial emotion perception at controlled and automatic processing levels (17, 18).

A common approach to examining the automaticity of affective processing is the masked priming paradigm in which a masked prime stimulus is presented briefly before a target stimulus (52). Individuals automatically recognize the affective valence of not consciously perceived words (53). Word primes can facilitate the recognition of subsequently presented facial expressions belonging to the same emotional category (54). The language-as-context hypothesis suggests that emotion words provide an internal context that helps constrain the multidimensional flow of environmental information (55, 56). It has been shown that gaze behavior in response to facial emotions can be influenced by emotional language. In the eye-tracking study by Provencio et al. (57), pairs of emotional and neutral faces were presented and primed by emotional words. Threat-related words led to an initial orienting away from angry faces, whereas depressive words triggered an early avoidance of angry faces in individuals high in paranoid beliefs (57). Since the prime words in this study were shown unmasked for 300 ms, avoidance of negative faces could represent conscious attention control. Furthermore, gaze behavior in response to emotional facial expressions was found to be affected by the implicit processing of emotional prosody (58): individuals look longer at faces that match the emotional quality of the prosody (e.g., happiness, anger, or fear). The effects of prosodic emotional primes on visual attention to faces were detected even seconds after the auditory information was no longer present (58).

In the present study, we explored whether emotion words (lexical primes) have an influence on the speed and duration subsequently presented facial emotions are looked at. Thus far, there has been little research on the automatic cross-domain (word–face) processing of emotional information and on attentional preferences for facial emotions in alexithymia. To this aim, we administered a free-viewing task in which four emotional facial expressions (i.e., happy, fearful, angry, and neutral) were displayed simultaneously. We analyzed early and late processes of attention allocation. It was expected that non-alexithymic individuals fixate facial emotions faster and longer when primed with a word of the same emotional quality than alexithymic individuals who exhibit reduced emotion-congruent priming. Finally, we assumed that non-alexithymic individuals manifest a more pronounced positivity or mood-protective bias than alexithymic individuals, i.e., they show a larger attentional preference for happy compared with neutral and negative faces.

## Materials and methods

### Participants

Participants were recruited through advertisements posted in public places and online on social media. A total of 166 interested individuals were interviewed *via* telephone by two trained doctoral medical students to assess the criteria for inclusion and exclusion. The interviewers were instructed and supervised by experienced clinical psychologists. The inclusion criteria were age between 18 and 35 years and German language. The exclusion criteria were the presence of a diagnosed mental disorder, visual impairment, neurological diseases, and the use of psychotropic medication. Individuals with psychotherapeutic, psychiatric, or neurological (inpatient or outpatient) treatments were excluded. Those who fit these criteria were asked to complete the 20-item Toronto Alexithymia Scale (TAS-20) (59, 60), a self-report measure assessing alexithymia. According to Bagby and Taylor (61), scores higher than 60 indicate alexithymia, while scores lower than 52 indicate the absence of alexithymia. Individuals who scored between 52 and 60 were excluded. The final sample consisted of 32 alexithymic and 46 non-alexithymic individuals. **Table 1** shows the demographic data for both groups. All participants had normal vision as examined with a Snellen eye chart. The Ethics Committee of the University of Leipzig, Medical School (DE/EKSN40), approved the study’s procedure. Informed written consent was obtained before the experiment. Participants were financially compensated upon completion of the study.

**Table 1:** Demographic and psychological test data of alexithymic and non-alexithymic individuals [means and SD (in parentheses) or frequency values].

Variable	Alexithymic individuals	Non-alexithymic individuals	*t*/*χ* ^2^	*p*
Age (years)	24.31 (5.11)	24.15 (4.56)	0.14	0.88
Sex (f/m)	18/14	26/20	0.01	0.98
School education (years)	12.03 (0.74)	12.10 (0.36)	-0.53	0.60
Intelligence (IQ, MWT-B)	106.41 (9.16)	111.63 (12.94)	-1.97	0.053*
TAS-20 (sum score)	67.91 (4.62)	38.57 (6.82)	21.17	<0.001***
STAI-S (item score)	1.95 (0.47)	1.78 (0.33)	1.84	0.07*
STAI-T (item score)	2.24 (0.47)	1.79 (0.42)	4.39	<0.001***
BDI-II (sum score)	11.03 (6.31)	7.13 (4.98)	3.05	0.003**

MWT-B, multiple-choice vocabulary test—version B; TAS-20, 20-item Toronto-Alexithymia Scale; STAI-S, State-Trait Anxiety Inventory—State version; STAI-T, State-Trait Anxiety Inventory—Trait version; BDI-II, Beck Depression Inventory II.

*p < 0.10; **p < 0.01; ***p < 0.001 (two-tailed).

### Questionnaires and tests

The *20-item Toronto Alexithymia Scale* (TAS-20) assesses three facets of alexithymia: difficulty identifying feelings, difficulty describing feelings, and externally oriented thinking (59, 60). Items are rated on a five-point scale. The total alexithymia score is the sum of responses to all 20 items with a range of possible scores from 20 to 100. Higher TAS-20 sum scores indicate higher alexithymia. In the overall sample, the Cronbach’s alpha for the TAS-20 sum score was 0.81.

The *State-Trait Anxiety Inventory* (STAI; German version) (62) is a self-report measure of state and trait anxiety. The STAI comprises 20 items, which are evaluated on a four-point Likert scale. The state version of the STAI measures the level of anxious feelings at the moment, whereas the trait version assesses stable interindividual differences in experiencing and evaluating situations as threatening. Cronbach’s alphas for the STAI state and STAI trait were 0.88 and 0.91, respectively, in the present sample.

The *Beck Depression Inventory* is a 21-question multiple-choice self-report scale (BDI-II; German version) (63) that measures the presence and severity of depressive symptoms such as negative cognitions, hopelessness, and physical symptoms during the preceding 2 weeks. In the total sample, Cronbach’s alpha for BDI-II was 0.81.

The *multiple-choice vocabulary intelligence test* (Mehrfachwahl-Wortschatz-Intelligenztest, MWT-B) (64) is a performance test that assesses aspects of general intelligence, specifically crystallized, verbal intelligence. The MWT-B consists of 37 items. Each item comprises four pronounceable pseudowords and one real word, which has to be recognized. MWT-B raw scores can be converted to IQ scores.

### Free-viewing task: stimuli and procedure

Facial stimuli consisted of 160 photographs of 40 white Swedish actors (20 women and 20 men) chosen from the Karolinska Directed Emotional Faces (65). Each actor clearly expressed four emotional facial expressions: happy, fearful, angry, and neutral. The target stimulus consisted of the simultaneous presentation of four different expressions of the same actor in a 2 × 2 matrix. The display size of each facial expression was 13 cm high × 9.5 cm wide. The vertical distance from the center of the photographs was 13.7 cm, while the horizontal distance from the photographs was 10.2 cm. All pictures were presented in their original color against a white background. Each actor was presented twice, resulting in a total of 80 trials. The position of the emotional faces in the 2 × 2 matrix of the target stimulus was changed in the second presentation.

Four words were used as lexical prime stimuli: “Freude” (in English, *happiness*), “Angst” (*anxiety*), “Wut” (*anger*), and “Neutral” (*neutral*).

Subjects were instructed in written form on the screen to view four pictures of faces. They were asked to view the images and to orient their gaze to the fixation cross immediately when it appeared. Participants were not informed about the presence of the prime stimuli. Each trial started with a fixation cross in the center of the screen (black cross against a white background) shown until a fixation of 1,000 ms. Subsequently, a lexical prime (happiness, anxiety, anger, or neutral) was presented very shortly for 50 ms, masked forward and backward by letter strings (i.e., sandwich-masked with a mask duration of 50 ms before and after the presentation of the prime). The sequence and duration of the prime and letter masks were equal to those used in two previous eye-tracking studies by our research group (66, 67). However, in these investigations, facial expressions served as primes and single faces were shown as target stimuli. In the present study, the letter strings consisted of 48 randomly chosen letters. The lexical primes were randomly presented with the restriction of no repetition of the same word on consecutive trials. The mask–lexical prime–mask sequence was then followed by the target stimulus for 5,000 ms (see **Supplementary Figure S1**). The overall duration of the free-viewing experiment was approximately 15 min.

### Eye tracking: apparatus and eye movement parameters

The eye-tracking experiment was conducted in a sound-attenuated room shielded from sunlight. Ceiling lighting produced stable illuminance conditions. Participants were seated in an adjustable chair approximately 65 cm away from the eye tracker. Stimuli were presented on a 24-in. LED monitor (resolution of 1,920 × 1,200, with 60 Hz refresh rate). The eye movements of the participants were recorded using a Tobii Pro Fusion eye tracker, fixed to the bottom of the monitor. The Tobii Pro Fusion captures gaze data at speeds up to 250 Hz per second. To calibrate the eye gaze position on the screen, a standard nine-point calibration procedure was conducted before the experimental task. Tobii Pro Lab software version 1.207.44884 (×64) (Tobii Technology, Stockholm, Sweden) was used to design the experiment, collect the eye-tracking data, and calculate the eye-tracking metrics. Statistical analysis was based on the 5,000-ms presentation period of the target stimuli. Therefore, four areas of interest (AOIs) were drawn around the four face images on each target stimulus.

We used the eye-tracking parameter *time to first fixation* as an indicator for processes of early attention allocation. Time to first fixation is defined as the latency (in milliseconds) from the start of the stimulus display until a specific AOI is first fixated upon. *Duration of fixation* was used as an indicator for sustained attention and attentional preference. Duration of fixation represents the sum of durations from all fixations (in milliseconds) that hit a specific AOI during a trial. Time to first fixation and duration of fixation were calculated for each AOI and each trial and then averaged for each participant.

### General procedure

The individual experimental sessions took place at the Department of Psychosomatic Medicine and Psychotherapy at the University of Leipzig. At the beginning of the study, participants completed the TAS-20, a sociodemographic questionnaire, and performed a vision test (using the Snellen eye chart). The above-mentioned tests and questionnaires were administered after the eye-tracking experiment in a fixed order: BDI-II, STAI state, MWT-B, and STAI trait.

### Statistical analyses

Two-sample *t*-tests and chi-square tests were administered to examine the differences between study groups in sociodemographic and psychological characteristics. The eye-tracking parameters *time to first fixation* and *fixation duration* were analyzed separately using a 4 × 4 × 2 mixed ANOVA with the prime word (happiness, anxiety, anger, or neutral) and facial target (happiness, fear, anger, or neutral expression) as within-subjects factors and study group (alexithymia *vs*. non-alexithymia) as a between-subjects factor. In case the assumption of sphericity was violated, the Greenhouse–Geisser correction was applied to adjust the degrees of freedom of the *F*-ratios (68). Follow-up tests were conducted to evaluate pairwise differences (Bonferroni-corrected pairwise comparisons). Results were considered significant at *p* < 0.05 (two-tailed). All calculations were administered using SPSS 29.0 (IBM Corp., Armonk, NY, USA).

## Results

### Sociodemographic and psychological variables

The descriptive statistics for the sociodemographic and psychological characteristics of the study groups are presented in **Table 1**. The study groups did not differ in age, sex distribution, and school education. However, alexithymic individuals tended to have higher state anxiety scores and lower intelligence scores than non-alexithymic individuals. Moreover, alexithymic individuals reported significantly more depressive symptoms and trait anxiety compared with non-alexithymic individuals (see **Table 1**).

### Time to first fixation

The mean times to first fixation as a function of emotional quality of the prime and target are shown for both study groups in **Table 2**. The 4 (prime condition) × 4 (target condition) × 2 (group) mixed ANOVA on time to first fixation revealed only a main effect of the target condition [*F*(2.68, 203.64) = 8.55, *p* < 0.001, *ηp* ^2^ = 0.10]. The results of the Bonferroni-adjusted pairwise comparisons indicated that time to first fixation was shorter for happy (1,504 ms) and fearful faces (1,511 ms) than for neutral faces (1,576 ms; *p*s ≤ 0.001). Moreover, the duration to first fixation was shorter for happy than for angry faces (1,551 ms; *p* < 0.05). Time to first fixation did not differ between the happy and fearful face conditions and between the angry and neutral face conditions. There were no significant effects of prime [*F*(2.69, 204.61) = 1.24, *p* = 0.29, *ηp* ^2^ = 0.016], prime × target [*F*(7.74, 587.96) = 0.74, *p* = 0.65, *ηp* ^2^ = 0.01], group × prime [*F*(2.69, 204.61) = 0.32, *p* = 0.79, *ηp* ^2^ = 0.004], group × target [*F*(2.68, 203.64) = 1.45, *p* = 0.23, *ηp* ^2^ = 0.019], and prime × target × group [*F*(7.74, 587.96) = 1.05, *p* = 0.39, *ηp* ^2^ = 0.014].

**Table 2:** Mean time to first fixation (in milliseconds) as a function of the emotional quality of the prime word and facial target for the study groups (means with standard deviations).

Prime word	Facial target	Alexithymic individuals Mean (SD)	Non-alexithymic individuals Mean (SD)
Happiness	Happiness	1,455 (438)	1,508 (322)
	Fear	1,457 (388)	1,543 (373)
	Anger	1,647 (413)	1,542 (373)
	Neutral	1,477 (370)	1,621 (413)
Anxiety	Happiness	1,486 (368)	1,525 (394)
	Fear	1,516 (390)	1,513 (371)
	Anger	1,539 (349)	1,604 (375)
	Neutral	1,574 (384)	1,664 (373)
Anger	Happiness	1,474 (441)	1,580 (395)
	Fear	1,526 (412)	1,527 (414)
	Anger	1,473 (310)	1,582 (354)
	Neutral	1,524 (361)	1,596 (360)
Neutral	Happiness	1,475 (459)	1,527 (407)
	Fear	1,454 (282)	1,552 (391)
	Anger	1,496 (348)	1,527 (359)
	Neutral	1,546 (404)	1,606 (374)

### Fixation duration

Participants’ mean fixation durations depending on the emotional quality of the prime and target and the study group are presented in **Table 3**. The 4 (prime condition) × 4 (target condition) × 2 (group) mixed ANOVA on fixation duration yielded only a main effect of the target condition [*F*(2.27, 172.44) = 5.26, *p* < 0.01, *ηp* ^2^ = 0.06] and a three-way interaction effect [*F*(5.63, 427.59) = 2.26, *p* < 0.05, *ηp* ^2 =^ 0.03]. The interaction effects group × target [*F*(2.27, 172.44) = 0.74, *p* = 0.49, *ηp* ^2^ = 0.01] and group × prime [*F*(2.61, 198.21) = 0.58, *p* = 0.60, *ηp* ^2^ = 0.008] were not significant. There were also no significant effects of prime [*F*(2.61, 198.21) = 0.37, *p* = 0.75, *ηp* ^2^ = 0.005] and prime × target [*F*(5.63, 427.59) = 0.76, *p* = 0.59, *ηp* ^2^ = 0.01].

**Table 3:** Mean fixation duration (in milliseconds) as a function of the emotional quality of the prime word and facial target for the study groups (means with standard deviations).

Prime word	Facial target	Alexithymic individuals Mean (SD)	Non-alexithymic individuals Mean (SD)
Happiness	Happiness	1,000 (319)	951 (226)
	Fear	1,010 (269)	916 (211)
	Anger	892 (233)	864 (249)
	Neutral	976 (240)	853 (231)
Anxiety	Happiness	964 (291)	987 (252)
	Fear	1,040 (259)	931 (211)
	Anger	894 (224)	871 (252)
	Neutral	965 (268)	835 (206)
Anger	Happiness	996 (273)	955 (215)
	Fear	998 (256)	954 (286)
	Anger	957 (237)	843 (216)
	Neutral	936 (251)	869 (232)
Neutral	Happiness	993 (297)	923 (202)
	Fear	968 (247)	953 (229)
	Anger	925 (223)	867 (240)
	Neutral	989 (261)	872 (245)

To further analyze the three-way interaction, separate two-factor ANOVAs (prime × target condition) were conducted on fixation duration for both groups. A 4 × 4 ANOVA based on the fixation times of alexithymic individuals yielded no main or interaction effects (all *p*s *>*0.10). A 4 × 4 ANOVA based on the fixation durations of non-alexithymic individuals showed a main effect of the target condition [*F*(2.31, 104.17) = 6.50, *p* ≤ 0.001, *ηp* ^2^ = 0.13]. No other significant effects were observed. The fixation durations as a function of the study group and target condition are shown in **Supplementary Figure S2**. The results of the Bonferroni-corrected pairwise comparisons indicated that happy faces (954 ms) were fixated on longer than neutral (857 ms) and angry faces (861 ms; *p*s < 0.05). Fearful faces (939 ms) were looked at longer than angry faces (*p* < 0.05). The fixation times did not differ between the happy and the fearful face conditions and between the angry and neutral face conditions. An additional 4 × 4 × 2 analysis of covariance (ANCOVA) was performed on the fixation durations with trait anxiety, depressive symptoms, intelligence, and state anxiety as covariates. The results of the 4 × 4 × 2 ANCOVA showed that the three-way interaction remained significant when controlling for the covariates [*F*(5.43, 390.73) = 2.20, *p* < 0.05, *ηp* ^2^ = 0.03].

Correlation analysis in the total sample (*N* = 78) showed that the TAS-20 score was significantly correlated with fixation duration on neutral faces (*r* = 0.29, *p* < 0.05), but not with fixation duration on happy, fearful, or angry faces (*r* = 0.08, 0.16, and 0.17, respectively).

## Discussion

In the present eye-tracking study, we compared the processes of lexical emotional priming and attention allocation to facial emotions between high alexithymic and non-alexithymic individuals. Our investigation should give insights into the automatic cross-domain processing of emotional information and attentional preferences in alexithymia. As could be expected, our alexithymic participants were characterized by increased anxiety and more depressive symptoms in comparison to the non-alexithymic participants. Alexithymia is known to be accompanied by heightened negative affectivity (12, 13), and its prevalence is increased in a number of mental disorders including depression (8). Alexithymia is assumed to augment the risk of depression through introspective emotion perception differences (14) and poor emotion regulation abilities (15, 69, 70).

Our data do not confirm the hypothesis that briefly shown lexical emotional information has an impact on subsequent attention allocation directing gaze toward facial expressions of the same emotional quality but suggest that masked emotion words have no influence on gaze direction. In contrast, the present results provide, at least in part, evidence in support of our second hypothesis that non-alexithymic individuals exhibit a more pronounced positivity or mood-protective bias than alexithymic individuals. That is, when non-alexithymic individuals looked at facial expressions, they fixated on happy faces longer than on neutral and angry faces. In addition, they looked longer at fearful than at angry faces. Thus, the present results provide evidence of attentional biases for positive and fearful social information in non-alexithymic individuals, but not in alexithymic individuals. Our results indicate a large effect size for emotional expression on fixation duration in non-alexithymic individuals. Alexithymic individuals instead showed no attentional preference for any expression category. The present data also suggest that alexithymia did not affect the early processes of attentional allocation as no group differences were detected in the speed of the first fixation on facial expressions. In general, alexithymic individuals did not look less at facial expressions compared with non-alexithymic individuals. On a descriptive level, the fixation duration was even longer for all emotion expressions in alexithymic than in non-alexithymic individuals. It appears that our alexithymic participants were motivated to follow the task instructions in the free-viewing experiment. The correlation analysis in the whole sample revealed that the TAS-20 alexithymia score was positively related to fixation duration on neutral faces. Thus, high alexithymia was accompanied by more attention allocation to neutral facial expressions. This correlation finding dovetails with our main ANOVA result: no attention advantage for emotional over neutral stimuli in alexithymia.

The observed late attentional orientation toward happy and fearful compared with neutral and angry faces in non-alexithymic participants appears to include, on the one hand, biases toward positive and fearful faces and, on the other hand, may also indicate an attentional avoidance of angry faces. Previous eye-tracking research has shown that non-alexithymic individuals manifest an attentional preference for positive stimuli (32–34) but also attentional biases to fear-related stimuli (40, 41). Thus far, only very few studies have investigated the deployment of attention using multi-stimulus free-viewing tasks showing angry and fearful (together with happy and neutral) faces at the same time. Bretthauer et al. (71) found an attentional pattern similar to ours in typically developing young adults in a multi-stimulus paradigm and specifically an attentional preference for facial fear and avoidance tendencies for facial anger. Free-viewing studies using face pairs also provided evidence for the avoidance of angry facial expressions and attentional bias toward fearful faces in non-alexithymic children, adolescents, and adults (43, 44). To avoid harmful consequences, fearful and angry faces require specific behavioral responses (71). Facial anger signals a proneness to engage in a conflict and is a sign of potential aggression and a direct threat (72, 73), whereas facial fear is an indicator of an indirect threat (danger in the environment) and the expresser’s loss of control (74). Angry faces have been shown to elicit avoidance-related behavior but fear faces facilitate approach behaviors in perceivers, supporting the idea that they represent affiliative stimuli (75). Avoidance of an angry face with eye gaze directed to the observer signals submissive behavior and serves as a protective function against aggressive attacks (76, 77). Against this background, an attentional preference for fearful compared with angry social signals, as found in our non-alexithymic participants, might be part of an implicit perceptual strategy to deal with threat information. Alexithymic individuals appear not to manifest such attention allocation in view of threat-related facial signals, which may negatively affect the initiation of appropriate actions and the handling of dangerous situations.

Motivational relevance can be defined as the significance of a situation or event to an individual’s goals and intentions (78). The motivational relevance of visual stimuli can guide gaze behavior at an early and late processing stage and be an important driver of prioritized visual processing (79). For example, McSorley et al. (40) showed that stimuli, which are more motivationally relevant to some individuals, i.e., unpleasant for spider-fearful individuals, can enhance early attentional processing. Giel et al. (80) observed that patients with anorexia nervosa exhibited no early attentional bias toward food pictures but avoided food pictures at a late phase of processing compared with non-anorexic controls. Patients with anorexia show a high motivation to restrict food intake in order to control body weight (81). Alexithymic individuals are characterized by a lack of subjective significance of emotions (5, 82). They believe that being in touch with one’s emotions is unimportant and that emotions are not useful in solving personal problems. Possibly, under free-viewing conditions, the low motivational relevance of emotions could be a factor contributing to the equal allocation of attention to emotional and neutral facial expressions at a late processing stage, which we observed in our alexithymic individuals.

In our study, non-alexithymic participants showed an attentional preference for happy faces. Such a positive bias can have mood-enhancing effects (35, 36). Guiding attention toward positive stimuli can protect against negative feeling responses to stressors and enhance reward perception (37, 38). A positive attentional bias can be considered as a form of antecedent emotion regulation (83) that increases the likelihood of positive feelings and reduces the probability of negative emotional experiences (39). Attentional processes are known to regulate emotional responses by tuning the filters for initial attention and subsequent processing (84, 85). Negatively biased attention to emotional information has been shown to be an important factor influencing the onset, maintenance, and recurrence of depression (86). The lack of attentional preference for positive stimuli, as observed in our alexithymic participants, might be a factor contributing to the development of depressive symptoms and negative affect. Beyond introspective differences in emotion perception (14) and poor emotion regulation (15, 69, 70), the lack of spontaneous attention deployment during the perception of positive stimuli could add to the vulnerability for depressive disorders in alexithymia. It is an interesting question whether attentional bias modification training (87) might be a useful therapeutic tool to promote gaze behavior toward positive social stimuli in alexithymic individuals.

In the present study, we explored whether masked emotional words direct subsequent gaze behavior toward emotion-congruent facial expressions. This assumption was made based on observations that people can identify the affective valence of not consciously perceived words (53) and that word primes can facilitate the recognition of facial expressions belonging to the same emotional quality (54). According to the language-as-context hypothesis (55), emotion words give an internal context, which helps constrain the multidimensional flow of environmental information. Against this background, it is plausible to assume that gaze behavior in response to facial emotions could be affected by emotional language. However, the data from our experiment suggest that lexical emotional information had no influence on gaze direction. The prime-related effects were consistently small in our study. Thus, the applied priming methodology did not produce relevant effects regarding gaze orientation. Future investigations on the topic should employ a different priming methodology and clarify whether a longer presentation time or an unmasking of the primes is necessary to achieve the expected effect or whether lexical emotional information does not direct attention toward emotion-congruent facial expressions at an automatic processing level.

Some limitations of the present study should be mentioned. These include the relatively small sample size of alexithymic individuals and the unequal sample sizes between study groups. Alexithymic individuals (fulfilling our inclusion and exclusion criteria) were much more difficult to recruit than non-alexithymic individuals. A high proportion of the project time was spent on the recruitment of alexithymic individuals. After 166 screening interviews, practical constraints led to the termination of the research project. In view of the small sample size, our results should be considered as preliminary. We calculated a *post-hoc* power analysis with the program G*Power 3.1 (88) for the observed group differences in attentional preference (fixation duration) for four facial targets (repeated measures ANOVA, *F*-tests, and within–between interactions). The achieved power in our study to detect a small to medium effect size (*f* = 0.15) was 0.58 given an alpha value of 0.05, a total sample size of 78 (with two groups and four measurements), a correlation between repeated measures of 0.50, and a non-sphericity correction of 0.34. This means that, if the true effect size is small to medium (*f* = 0.15), only approximately 6 out of 10 studies should produce a significant result. The estimated power of 0.58 is not satisfactory, so we recommend larger sample sizes to compare alexithymic and non-alexithymic individuals in future eye-tracking research examining spontaneous attention allocation to (four) emotional faces. Another limitation of our study was the selection of the alexithymia sample. We included only individuals without a mental (or neurological) disorder, which restricts the generalizability of the results. In addition, our study participants were young and well-educated individuals, which further limits the generalizability of our findings.

As mentioned in the *Introduction*, previous eye-tracking research based on free viewing (45–47) yielded rather inconsistent results, suggesting that specific facets of alexithymia might be related to the late avoidance of sad stimuli (47) or early avoidance of and late sustained attention toward fear stimuli (46). In all these studies, emotional scenarios were administered as stimuli; in contrast, emotional facial expressions were applied in our study, so a direct comparison of results is difficult. Moreover, none of the prior investigations examined the effect of high levels of alexithymia on attention to emotional information, which further decreases the comparability of results between studies. Although the dimensional approach in alexithymia research has, undoubtedly, its merits and scientific utility (16), we advocate not to neglect the effects of high levels of alexithymia on emotion perception. Some impairments in late processes of naturalistic attention allocation may become apparent only in high alexithymia. All in all, this study adds to our understanding of how alexithymic individuals attend to social–emotional signals in their environment and proposes tentative hypotheses regarding how alexithymia could be linked to negative affect and emotional disorders. The present alexithymia study focused on sustained attention allocation to facial emotions analyzing faces as a whole and aggregated fixation data across time. A recent eye-tracking study (89) revealed that alexithymia in autistic and non-autistic individuals is related to the atypical visual exploration of the eyes during emotion face perception. The findings of Cuve et al. (89) should motivate future studies in the field to focus more on the eyes as a central facial feature and to use multilevel polynomial modeling strategies to analyze the spatiotemporal dynamics of eye gaze in alexithymia.

## Data availability statement

The raw data supporting the conclusions of this article will be made available by the authors, without undue reservation.

## Ethics statement

The studies involving humans were approved by the Ethics Committee at the University of Leipzig, Medical Faculty (DE/EKSN40). The studies were conducted in accordance with the local legislation and institutional requirements. The participants provided their written informed consent to participate in this study.

## Author contributions

CS: Data curation, Formal analysis, Investigation, Writing – original draft. DH: Writing – review & editing. VG: Writing – review & editing. AK: Conceptualization, Writing – review & editing. MR: Writing – review & editing. TS: Conceptualization, Data curation, Formal analysis, Supervision, Writing – review & editing. CB: Conceptualization, Data curation, Formal analysis, Investigation, Writing – review & editing.
