# Supplementary material for: Deployment of attention to facial expressions varies as a function of emotional quality—but not in alexithymic individuals
Source: Front Psychiatry. 2024 Mar 6;15:1338194. doi: 10.3389/fpsyt.2024.1338194 (PMC10950908; doi:10.3389/fpsyt.2024.1338194)
Supplement: Supplementary file 1 [file DataSheet_1.pdf]

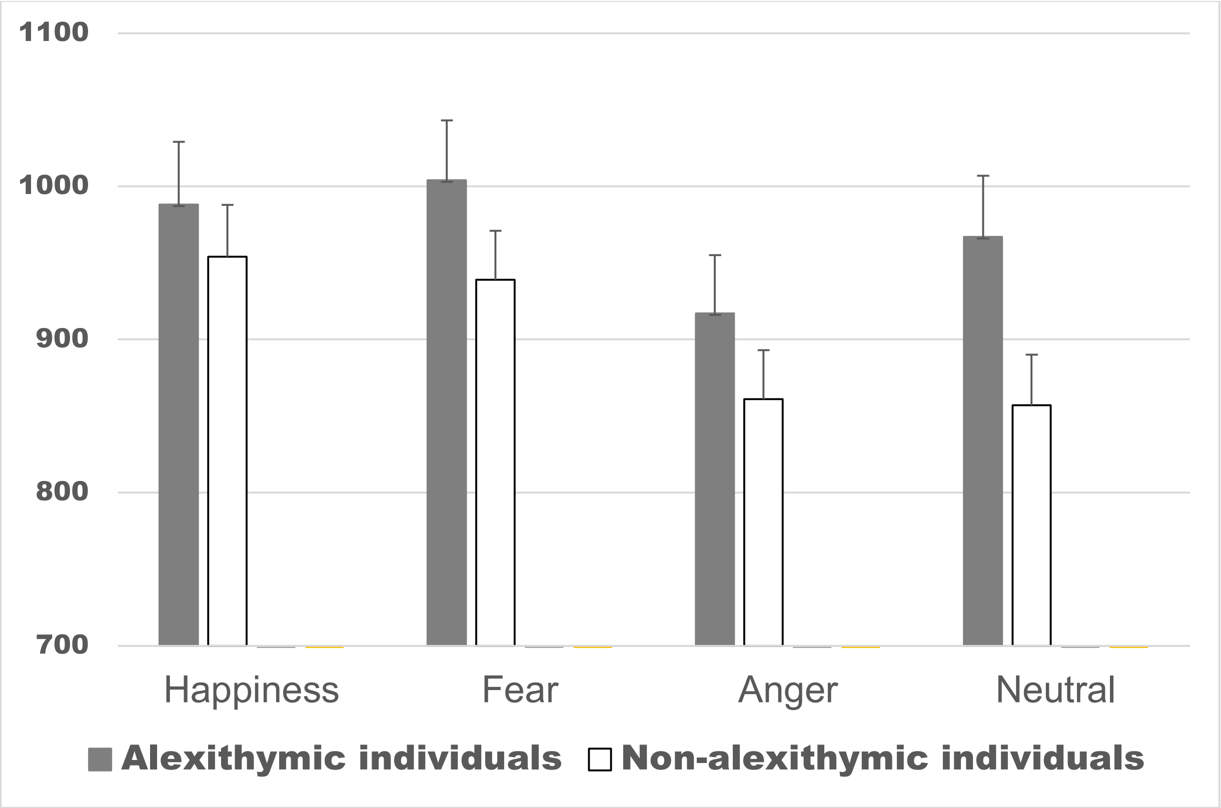

Figure 2: Mean fixation duration (in ms) as a function of emotional quality of facial target for study groups (means with standard error).
