# Supplementary figures and images for: Deployment of attention to facial expressions varies as a function of emotional quality—but not in alexithymic individuals
Source: Front Psychiatry. 2024 Mar 6;15:1338194. doi: 10.3389/fpsyt.2024.1338194 (PMC10950908; doi:10.3389/fpsyt.2024.1338194)

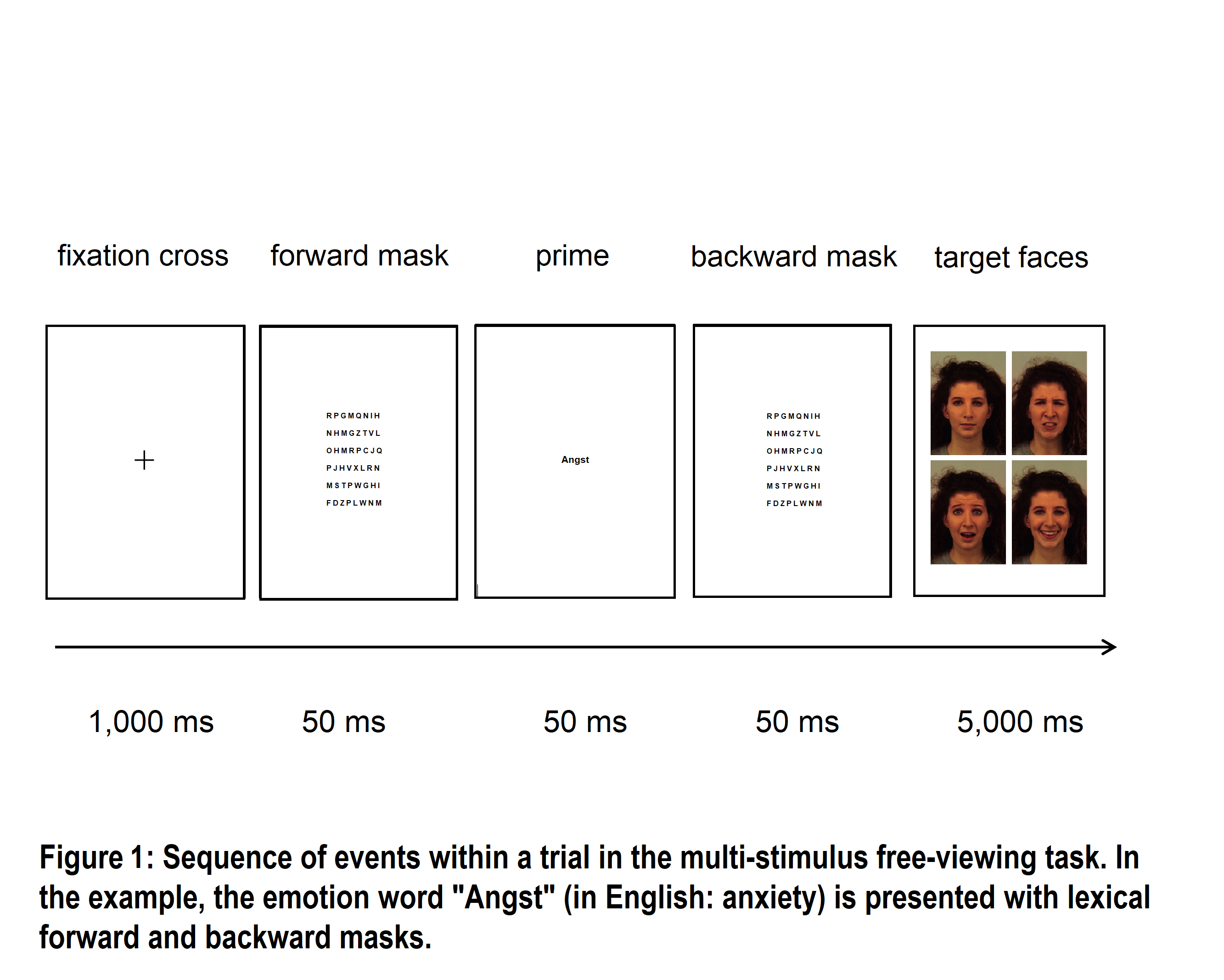

Supplement: Supplementary file 2 [file Image_1.tif]
